# New insights into the relationship between the average nucleotide identity and the digital DNA–DNA hybridization values in the genus *Amycolatopsis* and *Amycolatopsis cynarae* sp. nov., a novel actinobacterium from the rhizosphere soil of *Cynara scolymus*, and proposal of *Amycolatopsis niigatensis* as a synonym of *Amycolatopsis echigonensis* based on comparative genomic analysis

**DOI:** 10.3389/fmicb.2024.1359021

**Published:** 2024-04-15

**Authors:** Aihua Deng, Li Fu, Ping Mo, Yaxi Zheng, Ting Tang, Jian Gao

**Affiliations:** ^1^Key Laboratory of Agricultural Products Processing and Food Safety in Hunan Higher Education, Hunan Provincial Engineering Research Center for Fresh Wet Rice Noodles, Science and Technology Innovation Team for Efficient Agricultural Production and Deep Processing at General University in Hunan Province, Hunan Provincial Key Laboratory for Health Aquaculture and Product Processing in Dongting Lake Area, Hunan Provincial Key Laboratory for Molecular Immunity Technology of Aquatic Animal Diseases, State Key Laboratory of Developmental Biology of Freshwater Fish, College of Life and Environmental Sciences, Hunan University of Arts and Science, Changde, Hunan, China; ^2^School of Life and Health Sciences, Hunan University of Science and Technology, Xiangtan, Hunan, China

**Keywords:** International Streptomyces Project, ANIm and dDDH, corresponding relationship, *Amycolatopsis cynarae* sp. nov., synonym, *Amycolatopsis niigatensis*, *Amycolatopsis echigonensis*

## Abstract

At present, it is widely believed that a 95–96% average nucleotide identity (ANI) value is equivalent to a 70% digital DNA–DNA hybridization (dDDH) value in the prokaryotic taxonomy. However, in the present study, comparative genome analysis of 29 pairs of *Amycolatopsis* type strains revealed that a 70% dDDH value did not correspond to a 95–96% ANI based on the MuMmer ultra-rapid aligning tool (ANIm) but approximately corresponded to a 96.6% ANIm value in the genus *Amycolatopsis*. Based on this corresponding relationship, phenotypic and chemotaxonomical characteristics, as well as phylogenetic analysis, an actinobacterial strain HUAS 11-8^T^ isolated from the rhizosphere soil of *Cynara scolymus*, was subjected to a polyphasic taxonomic characterization. Based on EzBioCloud alignment, it was found that strain HUAS11-8^T^ had the 16S rRNA gene similarities of 99.78% with *A. rhizosphaerae* JCM 32589^T^, 97.8% with *A. dongchuanensis* YIM 75904^T^, and < 97.8% sequence similarities to other *Amycolatopsis* species. Phylogenetic analysis of 16S rRNA gene sequences and whole-genome sequences revealed that strain HUAS 11-8^T^ was closely related to *A. rhizosphaerae* JCM 32589^T^. ANIm and dDDH values between strains HUAS 11-8^T^ and *A. rhizosphaerae* JCM 32589^T^ were 96.3 and 68.5%, respectively, lower than the 96.6 and 70% thresholds recommended for the delineation of a novel *Amycolatopsis* species. Consequently, strain HUAS 11-8^T^ should represent a novel *Amycolatopsis* species, for which the name *Amycolatopsis cynarae* sp. nov. (type strain HUAS 11-8^T^ = MCCC 1K08337^T^ = JCM 35980^T^) is proposed. Furthermore, based on comparative genomic analysis and rule 42 of the Prokaryotic Code, we propose that *Amycolatopsis niigatensis* is a later heterotypic synonym of *Amycolatopsis echigonensis*.

## 1 Introduction

The genus *Amycolatopsis*, belonging to the family Pseudonocardiaceae of the order Pseudonocardiales, was initially described using specific methods (Lechevalier et al., 1986) and then emended by Lee (2009) and Tang et al. (2010). At the time of writing, this genus included more than 80 species with validly published and correct names.^1^ The members of the genus *Amycolatopsis* are distributed in various environments such as peat swamp forest soil (Teo et al., 2020, 2021), deep-sea sediment (Zhang et al., 2016), arid soil (Tan et al., 2006; Zucchi et al., 2012; Busarakam et al., 2016), plant tissues (Wang et al., 2020; Tedsree et al., 2021), animals, and humans (Labeda et al., 2003; Huang et al., 2004). In the past 10 years, *Amycolatopsis* strains have gained widespread attention due to their potential in antibiotic production, bioremediation, bioconversion, and biodegradation processes (Dávila Costa and Amoroso, 2014; Adamek et al., 2018). Therefore, it is of great practical significance to search for *Amycolatopsis* strains, especially novel *Amycolatopsis* species.

Recently, in a survey on the diversity of actinobacteria from the rhizospheric soil of different plants, 100 s of strains were isolated using different media. Interestingly, the conclusions were contradictory if the taxonomic status of strain HUAS 11-8^T^, one of all those strains mentioned above, was described using the different classification criteria. In addition, in the course of analyzing the relatedness between average nucleotide identity based on the MuMmer ultra-rapid aligning tool (ANIm) and digital DNA–DNA hybridization (dDDH) in the genus *Amycolatopsis*, we found that *Amycolatopsis niigatensis* and *Amycolatopsis echigonensis* should be of the same genomic species. Thus, the main aims of the present study are to (1) elucidate the reasons for the abovementioned contradictory results, (2) evaluate the taxonomic status of strain HUAS 11-8^T^ using a polyphasic taxonomic approach, and (3) clarify the taxonomic relation between *A. niigatensis* and *A. echigonensis* based on comparative genomic analysis.

## 2 Materials and methods

### 2.1 Genome data used to analyze the relationship between ANIm and dDDH values in the genus *Amycolatopsis*

A total of 29 genomes from type strains of *Amycolatopsis* species with validly published names were downloaded from the GenBank database. All genomes used in this study must meet the criteria of >95% completeness and < 5% contamination in order to obtain more reliable analysis results. The quality analysis and GenBank assembly of genomes are shown in Supplementary Table S1.

As Meier-Kolthoff and Göker (2019) proposed, when the ANI value between two genomes is more than 90%, ANIm can provide more credible results with respect to ANI based on the BLAST algorithm (ANIb). Thus, the ANIm value was selected for comparative analysis in this study. The ANIm and dDDH values were calculated by using the JSpeciesWS online service (Richter et al., 2016) and the Genome-to-Genome Distance Calculator with Formula 2 (Meier-Kolthoff et al., 2013), respectively. The coherence analysis between ANIm and dDDH values was performed using these methods (Hu et al., 2022).

### 2.2 Evaluation of the taxonomic status of strain HUAS 11-8^T^

#### 2.2.1 Isolation and maintenance of strain HUAS 11-8^T^

Strain HUAS 11-8^T^ was isolated from the rhizosphere soil of *Cynara scolymus*, which was collected in Changde city of Hunan Province, China (29.20201^°^ N 111.98113^°^ E), as described by Mo et al. (2017). The purified strain HUAS 11-8^T^ was prepared for short-term preservation on Reasonerʼ2A (Reasoner and Geldreich, 1985) slopes at 4°C and suspended in a sterile 30% (w/v) glycerol solution for long-term conservation at −80°C. The type strain *Amycolatopsis rhizosphaerae* JCM 32589^T^ was purchased from the Japan Collection of Microorganisms (JCM). Strains HUAS 11-8^T^ and *A. rhizosphaerae* JCM 32589^T^ were tested under the same conditions.

#### 2.2.2 Genome sequencing and phylogenetic analysis

The genome sequencing of strain HUAS 11-8^T^ was completed by using a Nanopore PromethION sequencing system at Wuhan Benagen Technology Co., Ltd. (Hubei, China). Genomic DNA extraction and PCR conditions of the 16S rRNA gene were carried out using the method described by Mo et al. (2023). The 16S rRNA gene of strain HUAS 11-8^T^ was amplified using universal primers (27F and 1492R) (Lane, 1991). The 16S rRNA gene sequence of strain HUAS 11-8^T^ was compared with the EzBioCloud database^2^ (Yoon et al., 2017). Closely related reference strains were downloaded and used for constructing phylogenetic trees using neighbor-joining (NJ) (Saitou and Nei, 1987), maximum-likelihood (ML) (Felsenstein, 1981), and maximum-parsimony (MP) (Kluge and Farris, 1969) with 1,000 bootstrap replications in MEGA 11 (Tamura et al., 2021). According to the result of 16S rRNA gene sequence analysis, the genome sequences of type strains that were closely related to strain HUAS 11-8^T^ were selected for reconstructing the phylogenomic tree using the Type (Strain) Genome Server. The ANIm and dDDH values between the genomes of strain HUAS 11-8^T^ and its relatives were calculated according to the aforementioned description. The gene prediction analysis and functional annotation of the genome of strain HUAS 11-8^T^ were performed by the NCBI Prokaryotic Genome Annotation Pipeline v4.4 and Rapid Annotation using Subsystem Technology v.2.0 (RAST^3^) (Aziz et al., 2008). The secondary metabolism biosynthetic gene clusters of strain HUAS 11-8^T^ and antibiotic resistance genes were analyzed using antiSMASH version 6.0.1^4^ and the Comprehensive Antibiotic Resistance Database (CARD^5^), respectively (Blin et al., 2011; Alcock et al., 2020). Clustered regularly interspaced short palindromic repeat sequences (CRISPRs) of strain HUAS 11-8^T^ were identified by CRISPR-case Finder^6^ (Makarova et al., 2019), and then the genomic islands were predicted by the Island Viewer 4 webserver (Claire et al., 2017).^7^

#### 2.2.3 Morphological, cultural, and physio-biochemical characteristics

Spore features of HUAS 11-8^T^ were observed by a light microscope (NE620, Ningbo Yongxin Optics) and scanning electron microscope (FEI-Quanta 450, America), using cultures grown on Reasonerʼ 2A after incubation for 21 days at 28°C. The cultural features of strains HUAS 11-8^T^ and *A. rhizosphaerae* JCM 32589^T^ were observed on various media, including Gauseʼs synthetic No. 1 medium (Atlas, 1993), Reasonerʼ 2A and ISP 2–7 media (Shirling and Gottlieb, 1966) for 21 days at 28°C. Color determinations, such as the aerial mycelium, the substrate mycelium, and diffusible soluble pigments, were delineated according to the methods of Shirling and Gottlieb (1966). Growth was carried out on tryptic soy broth (TSB) medium for 14 days at temperatures (4, 10, 15, 20, 22, 25, 27, 30, 35, 37, 40, 45, 50, and 55°C), pH (2.0–12.0, at intervals of 1.0 pH unit), and concentrations of NaCl (0–15%, w/v, at an interval of 1% w/v). The following tests, i.e., carbon and nitrogen source utilization, starch hydrolysis, gelatin liquefaction, nitrate reduction, and Tweens (20, 40, 60, and 80) degradation, were performed according to the methods described by Xu et al. (2007).

#### 2.2.4 Chemotaxonomical characteristics

Biomass for chemotaxonomic analysis was collected by centrifugation after culturing for 5–7 days at 28°C in TSB in shake flasks. The cellular fatty acids of strains HUAS 11-8^T^ and *A. rhizosphaerae* JCM 32589^T^ were detected as described by Sherlock MIDI protocol (Sherlock Microbial Identification System, version 6.0B) (MIDI, 2005), which were carried out by the Marine Culture Collection of China (MCCC). The diaminopimelic acid isomers in the cell wall peptidoglycan were separated by thin-layer chromatography and analyzed using a solution of ninhydrin in acetone (Hasegawa et al., 1983). The whole-cell sugars were analyzed as described by Lechevalier and Lechevalier (1970). Polar lipids and menaquinones were extracted and analyzed using the method described by Ruan and Huang (2011).

## 3 Results and discussion

### 3.1 Relationship between ANIm and dDDH values in the genus *Amycolatopsis*

**Figure 1:**
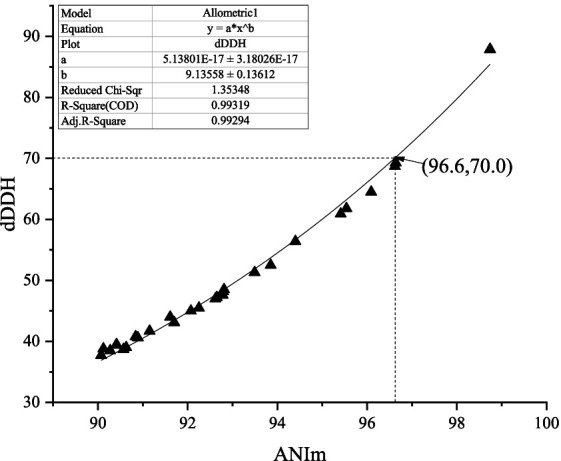
Correlations between ANIm and dDDH from the 29 pairs of *Amycolatopsis* species.

Over the past 50 years, the traditional DNA–DNA hybridization (DDH) technology has played a key role in the classification and identification of *bacteria* and *archaea*. However, there is a large amount of evidence that this technology has its limitations, such as being labor-intensive, error-prone, and difficult to generate cumulative databases. Thus, there has been an urgent need to look for an alternative standard (Stackebrandt et al., 2002; Gevers et al., 2005). At present, as alternative standards based on the genome level, ANI values of 95–96% and dDDH values of 70% have generally acted as a gold standard for bacterial species delineation (Richter and Rosselló-Móra, 2009; Auch et al., 2010). Nevertheless, during our search for novel actinobacteria capable of producing bioactive compounds, we found that strain HUAS 11-8^T^, one of all actinomycete strains isolated, should belong to a new *Amycolatopsis* species according to the 68.5% dDDH value between the strain and its relative, but this strain should belong to a known *Amycolatopsis* species according to the 96.3% ANIm value between the strain and its relative. What are the reasons for this contradictory phenomenon? To resolve this question, we first calculated the ANIm values between all validly published *Amycolatopsis* species whose genomes were available. Then, the dDDH values between all pairs of strains, whose ANIm values were ≥ 90%, were calculated for subsequent analysis. Finally, the dDDH and ANIm values of a total of 29 pairs of *Amycolatopsis* species (including synonyms) were randomly selected for correlation analysis (Supplementary Table S2). As shown in Figure 1, the dDDH value revealed an extremely high correlation (R^2^ = 0.99319) with the ANIm value based on an exponential regression model. However, a 70% dDDH value was not equivalent to a 95–96% ANIm value, but to an ANIm value of approximately 96.6%. Thus, the contradiction above can be well explained based on this corresponding relationship. At present, in addition to AINm, ANIb is also a mainstream ANI computing model. Then, how does dDDH correspond to ANIb in the genus *Amycolatopsis*? As shown in Supplementary Figure S1, a 70% dDDH value approximately corresponded to a 95.8% ANIb value, which is in the middle of 95–96%. Although the dDDH value also showed an extremely high correlation (>0.99) with the ANIb value based on an exponential regression model, this corresponding relationship did not well explain the contradictory phenomena above. Furthermore, the conclusions were completely consistent if all currently known *Amycolatopsis* species with validly published and correct names were delineated using a 96.6% ANIm value or 70% dDDH value (data not shown). Thus, we recommended that a 96.6% ANIm value could act as the threshold for delineating *Amycolatopsis* species.

### 3.2 Evaluation of the taxonomic status of strain HUAS 11-8^T^

#### 3.2.1 Genome analysis

The genome sequence size of strain HUAS 11-8^T^ is 7,474,574 bp with a DNA G + C content of 70.3%. A total of 7,267 genes (7,092 coding genes, 74 RNA genes, and 101 pseudogenes) and 7,193 CDSs (7,092 CDSs with protein and 101 CDSs without protein) were predicted. The analysis of the genome of strain HUAS 11-8^T^ by the RAST Server revealed 316 subsystems that could be classified into 23 categories, and the subsystem coverage was 19%. The represented subsystem features identified were “Amino Acids and Derivatives” (378 CDSs), “Carbohydrates” (368 CDSs), “Fatty Acids, Lipids, and Isoprenoids” (214 CDSs), “Cofactors, Vitamins, Prosthetic Groups, Pigments” (213 CDSs), “Protein Metabolism” (173 CDSs), “DNA Metabolism” (121 CDSs), “Respiration” (109 CDSs), “Nucleosides and Nucleotides” (92 CDSs), “Metabolism of Aromatic Compounds” (91 CDSs), “Virulence, Disease and Defense” (63 CDSs), “Miscellaneous” (51 CDSs), “RNA Metabolism” (51 CDSs), “Stress Response” (44 CDSs), “Cell Wall and Capsule” (39 CDSs), “Membrane Transport” (35 CDSs), “Nitrogen Metabolism” (35 CDSs), “Phosphorus Metabolism” (32 CDSs), “Regulation and Cell signaling” (22 CDSs), “Sulfur Metabolism” (11 CDSs), “Iron acquisition and metabolism” (8 CDSs), “Dormancy and Sporulation” (7 CDSs), “Potassium metabolism” (6 CDSs), and “Secondary Metabolism” (3 CDSs). RAST revealed that strain HUAS 11-8^T^ comprised lots of putative genes known to be associated with the abilities of dealing with harsh environmental conditions found in plant-associated environments, such as osmotic stress, oxidative stress, detoxification, stress response-no subcategory (SigmaB stress response regulation, dimethylarginine metabolism, and bacterial hemoglobins), and periplasmic stress. Three genes (*Streptomyces venezuelae* rox, vanR gene in vanO cluster, and *Mycobacterium tuberculosis* folC with mutation conferring resistance to para-aminosalicylic acid) of strain HUAS 11-8^T^ related to antibiotic resistance were recognized by CRAD analysis, which might confer resistance to rifamycin antibiotic, glycopeptide antibiotic, and salicylic acid antibiotic. The potential secondary metabolite biosynthetic gene clusters in strain HUAS 11-8^T^ were analyzed by antiSMASH, and 16 gene clusters were annotated. The three main biosynthetic gene clusters were aryl polyene, non-ribosomal peptide synthetase (NRPS), and type I polyketide synthase (PKS) [T1PKS]. The two T1PKS gene clusters had 96 and 8% similarities to macrotermycins and A54145, respectively. Meanwhile, nine CRISPR repeats of strain HUAS 11-8^T^ were identified in the genome. In total, 10 genetic islands with a size range from 4,724 to 30,712 bp were identified in the genome of strain HUAS 11-8^T^.

#### 3.2.2 Phylogenic analysis

**Figure 2:**
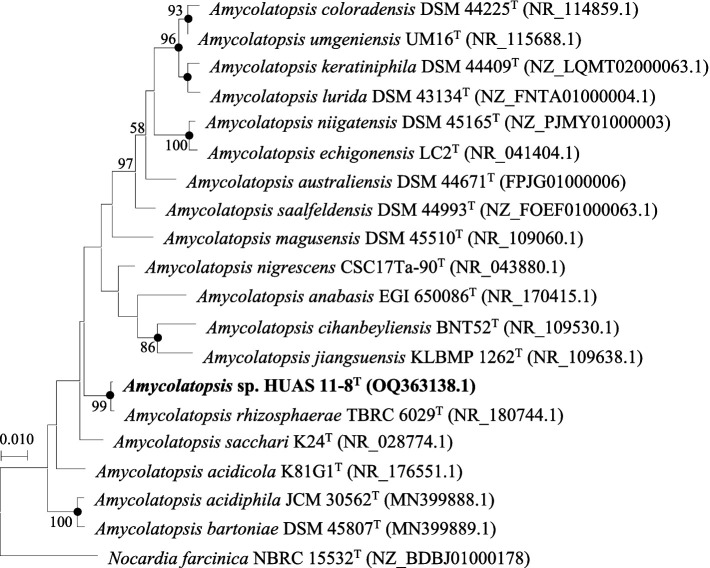
Maximum-likelihood phylogenetic tree based on 16S rRNA gene sequences showing the relationship between selected species of the genus *Amycolatopsis*. *Nocardia farcinica* NBRC 15532^T^ was used as an outgroup. Bootstrap percentages over 50% derived from 1,000 replications are shown at the nodes. Dots indicate branches also recovered in the neighbor-joining and maximum-parsimony trees. Bar, 0.010 substitutions per site.

**Figure 3:**
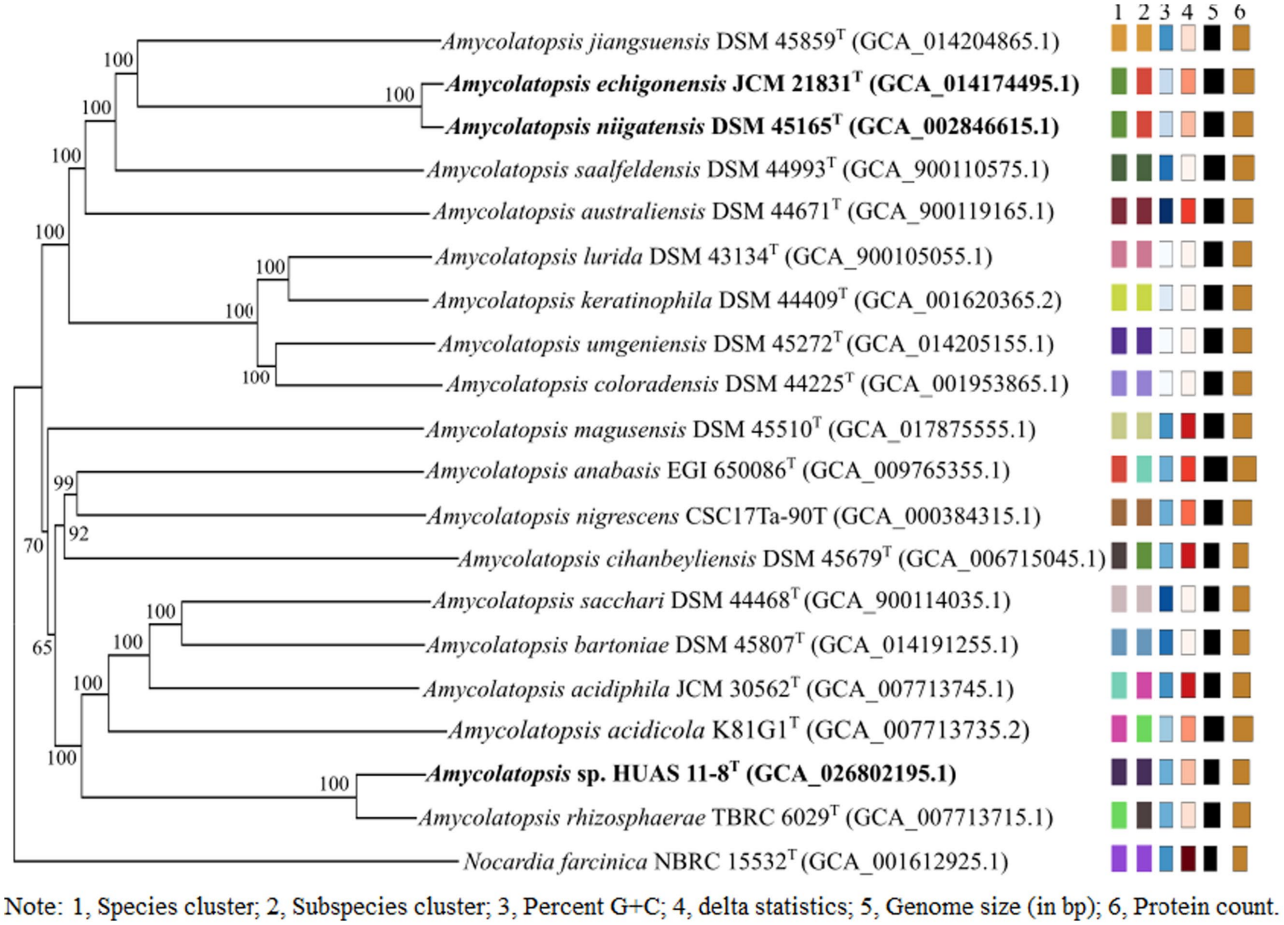
Phylogenetic tree based on whole-genome sequences of HUAS 11-8^T^ and related reference strains. Tree inferred with FastME 2.1.6.1 (Vincent et al., 2015) from GBDP distances calculated from genome sequences. The branch lengths are scaled in terms of the GBDP distance formula d5. The numbers above branches are GBDP pseudo-bootstrap support values >60% from 100 replications, with an average branch support of 96.0%. The tree was rooted at the midpoint (Farris, 1972).

Based on EzBioCloud perform alignment, it was found that strain HUAS11-8^T^ had 16S rRNA gene similarities of 99.78% with *A. rhizosphaerae* JCM 32589^T^, 97.8% with *A. dongchuanensis* YIM 75904^T^, and < 97.8% sequence similarities to other *Amycolatopsis* species. An ML phylogenetic tree based on 16S rRNA gene sequence demonstrated that strain HUAS 11-8^T^ was most closely related to *A. rhizosphaerae* JCM 32589^T^ (Figure 2). The relationship between HUAS 11-8^T^ and *A. rhizosphaerae* JCM 32589^T^ also appeared in the NJ and MP trees (Supplementary Figures S2, S3). This topological structure was further supported by the results of phylogenomic analysis (Figure 3). Nevertheless, the ANIm and dDDH values between strains HUAS 11-8^T^ and *A. rhizosphaerae* JCM 32589^T^ were 96.3 and 68.5%, respectively, lower than the 96.6 and 70% thresholds recommended for the delineation of a novel *Amycolatopsis* species. Furthermore, this result was further confirmed by phenotypic and chemotaxonomic differences between strains HUAS 11-8^T^ and *A. rhizosphaerae* JCM 32589^T^ (Table 1). For example, the spore chains of strain HUAS11-8^T^ are branched, and the spores are oval, spherical, and short-rod. While *A. rhizosphaerae* JCM 32589^T^ produces long spore chains and spherical spores. In addition, the dominant menaquinones are MK-9 (H_4_), MK-9 (H_2_), and MK-9 (H_6_). Galactose, ribose, and xylose were detected as the whole-cell reducing sugars in strain HUAS11-8^T^. The dominant menaquinone of strain *A. rhizosphaerae* JCM 32589^T^ is MK-9 (H_4_, _6_, _8_) and MK-10 (H_2_, _6_). Galactose and arabinose were detected as the whole-cell reducing sugars in strain *A. rhizosphaerae* JCM 32589^T^.

**Table 1:** Differential characteristics of strains HUAS 11-8^T^ and *A. rhizosphaerae* JCM 32589^T^.

Characteristics	1	2
Spore chain	Branch	Long chains
Spore surfaces	Smooth	Smooth
Spore shape	Oval, spherical, short-rod	Spherical
Nitrate reduction	−	+
Hydrolysis of tweens (60 and 80)	−	+
Growth temperature (°C)	20–35	15–45
Tolerance to NaCl (%, w/v)	0–3%	0–5%
The pH range for growth	6.0–9.0	5.0–10.0
**Sole carbon source utilization**		
Glucose	+	−
Inositol	+	−
l-Arabinose	+	−
Sucrose	+	−
**Sole nitrogen source utilization**		
l-Arginine	+	−
l-Histidine	−	+
l-Hydroxyproline	−	+
l-Ornithine	−	+
l-Phenylalanine	+	−
l-Tyrosine	−	+
l-Valine	+	−
Menaquinones	MK-9 (H_2_, _4_, _6_)	MK-9 (H_4_, _6_, _8_) MK-10 (H_2_, _6_)
Cell-wall diamino acid	*meso*-DAP	*meso*-DAP
Whole-cell sugars	Galactose, ribose, xylose	Galactose, arabinose

1, strain HUAS 11-8^T^; 2, *Amycolatopsis rhizosphaerae* JCM 32589^T^. +, positive; −, negative. All strains were positive for the hydrolysis of starch and negative for the hydrolysis of Tweens (20 and 40). All strains can utilize cellobiose, d-ribose, mannitol, and xylose as sole carbon sources; but not for d-galactose, d-mannose, fructose, lactose, and l-rhamnose. All strains can utilize l-glutamine, l-leucine, and l-serine as sole nitrogen sources; but not for l-Alanine, l-asparagine, l-cysteine, l-glycine, l-proline, and methionine. All phenotypic data were determined in this study.

#### 3.2.3 Morphological, physiological, and chemotaxonomic characteristics

**Figure 4:**
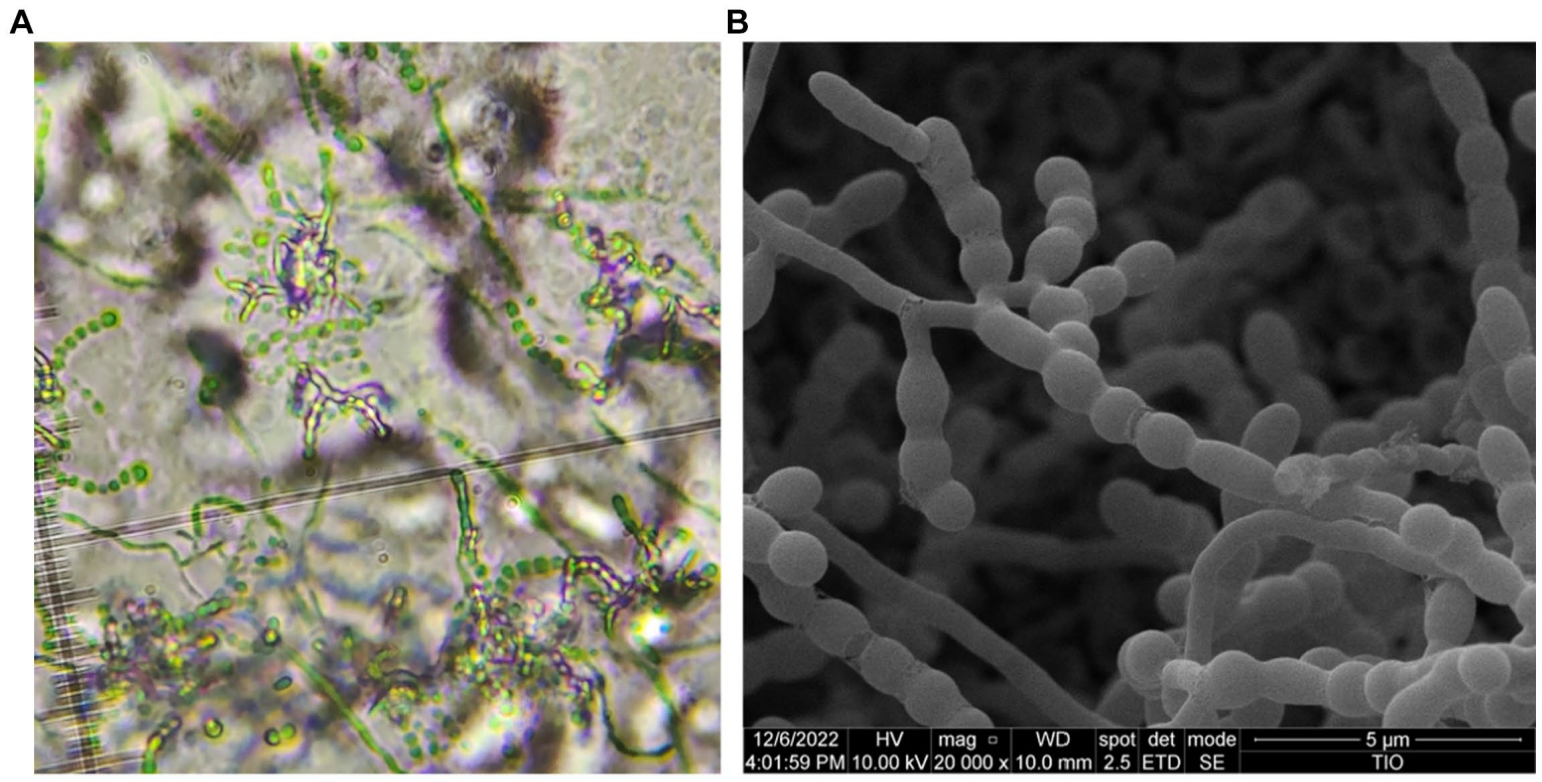
Optical microscope **(A)** and scanning electron microscope **(B)** images of strain HUAS 11-8^T^ grown on Reasonerʼ2A after incubation for 21 days at 28°C.

Morphologically, strain HUAS 11-8^T^ produced white substrate hyphae and aerial mycelia that differentiated into spore chains on Reasonerʼ2A medium after incubation for 21 days at 28°C. Spore chains were branched, and spore surfaces were smoothed (Figure 4). This strain was observed to grow well on Gauseʼs synthetic No. 1 medium, Reasonerʼ2A, and ISP 2–7 serial media (Supplementary Table S3). Growth was observed at 20–35°C (optimum, 30°C), at pH 6.0–9.0 (optimum, pH 7.0), and in the presence of 0–3% of NaCl (optimum, 0–1%). Detailed physiological and biochemical characteristics are provided in the species description. The dominant menaquinones were MK-9(H_4_) (75.2%), MK-9(H_2_) (20.6%), and MK-9(H_6_) (2.1%). The strain was found to contain *meso*-diaminopimelic acid as the cell wall amino acid. Galactose, ribose, and xylose were detected as whole-cell reducing sugars. The major fatty acids (≥10%) were *iso*-C_16:0_ (30.5%), C_16:0_ (10.8%), and C_17:1_ ω6c (10.0%). The detailed fatty acid composition is shown in Supplementary Table S4. The polar lipid pattern consisted of diphosphatidylglycerol, phosphatidylcholine, phosphatidylethanolamine, phosphatidylglycerol, and phosphatidylinositol (Supplementary Figure S4). All these morphological and chemotaxonomic data were consistent with the assignment of strain HUAS 11-8^T^ to the genus *Amycolatopsis*.

In conclusion, based on phenotypic, chemotaxonomic, and genotypic data, strain HUAS 11-8^T^ could be distinguished from *A. rhizosphaerae* JCM 32589^T^. Therefore, it is concluded that strain HUAS 11-8^T^ represents a novel species of the genus *Amycolatopsis*, for which the name *Amycolatopsis cynarae* sp. nov. is proposed.

### 3.3 A proposal of *Amycolatopsis niigatensis* as a later heterotypic synonym of *Amycolatopsis echigonensis*

In order to determine the corresponding relationship between ANIm and dDDH values in the genus *Amycolatopsis*, we first analyzed the 16S rRNA gene sequence similarity between each validly published *Amycolatopsis* species and other validly published *Amycolatopsis* species. As a result, it was found that *A. echigonensis* JCM 21831^T^ shared ≥98.7% sequence similarities to *A. niigatensis* DSM 45165^T^, *A. halotolerans* NRRL B-24428^T^, *A. albidoflavus* NRRL B-24149^T^, *A. rubida* NRRL B-24150^T^, *A. circi* S1.3^T^, *A. nivea* CFH S0261^T^, *A. equina* SE (8)3^T^, *A. dendrobii* DR6-1^T^, and *A. hippodromi* S3.6^T^, respectively (Supplementary Tables S5, S6). According to the proposal of Stackebrandt and Ebers (2006), a 16S rRNA gene sequence similarity threshold range of 98.7–99% is the point at which DNA–DNA reassociation experiments should be mandatory for testing the genomic uniqueness of a novel isolate. Thus, it is necessary to evaluate DNA–DNA relatedness between *A. echigonensis* JCM 21831^T^ and its relatives. The result showed that the ANIm and dDDH values between *A. echigonensis* JCM 21831^T^ and *A. niigatensis* DSM 45165^T^ were 98.7 and 87.9%, respectively, much higher than the 96.6 and 70% thresholds recommended for the delineation of a novel *Amycolatopsis* species, suggesting that *A. echigonensis* JCM 21831^T^ and *A. niigatensis* DSM 45165^T^ belonged to the same genomic species. This result was further supported by the phylogenomic analysis (Figure 3). In addition, the ANIm/dDDH values between *A. echigonensis* JCM 21831^T^ and type strains *of* other four species (*A. rubida*, *A. circi*, *A. nivea*, and *A. dendrobii*) were 92.5%/45.8, 92.2%/45.5, 92.6%/47.0, and 92.7%/47.2%, respectively, much less than the 96.6 and 70% thresholds recommended for the delineation of a novel *Amycolatopsis* species. Unfortunately, due to the unavailability of genomic data for type strains of *A. halotolerans*, *A. albidoflavus*, *A. equina*, and *A. hippodrome*, we could not evaluate DNA–DNA relatedness between *A. echigonensis* JCM 21831^T^ and them. Whereas, there is evidence that a *gyrB* genetic distance of >0.02 or a *recN* genetic distance of >0.04 between two *Amycolatopsis* strains is proposed to provide a good indication that they belong to different species (Everest and Meyers, 2009; Everest et al., 2011). As shown in Supplementary Table S5, *gyrB* or *recN* genetic distances between *A. echigonensis* JCM 21831^T^ and type strains of the above-mentioned four *Amycolatopsis* species were well over the thresholds recommended for assessing quickly whether an isolate is worthy of full taxonomic characterization.

Based on the analysis above and rule 42 of the Prokaryotic Code (Oren et al., 2022), we propose that *Amycolatopsis niigatensis* (Ding et al., 2007) (Approved Lists, 2007) (Euzeby, 2007) is a later synonym of *Amycolatopsis echigonensis* (Approved Lists, 2007).

## 4 Description

### 4.1 Description of *Amycolatopsis cynarae* sp. nov

*Amycolatopsis cynarae* [cy.na’rae. N.L. fem. n. Cynara, genus name of artichoke; N.L. gen. Fem. n. cynarae, of *Cynara* (*Cynara scolymus* L.)].

Aerobic, Gram-positive actinobacterium that forms white substrate hyphae and aerial mycelia that differentiate into branched spore chains consisting of smooth-surfaced oval, spherical, and short-rod spores on Reasonerʼ2A medium. Good growth is observed on all tested media. No diffusible pigment is produced on all tested media. Positive for the hydrolysis of starch, but negative for the hydrolysis of Tweens (20, 40, 60, and 80). Growth occurs at 20–35°C (optimum, 30°C), at pH 6.0–9.0 (optimum, pH 7.0), and in the presence of 0–3% of NaCl (optimum, 1%). Cellobiose, d-ribose, glucose, inositol, l-arabinose, mannitol, sucrose, and xylose can be utilized as sole carbon sources, but not for d-galactose, d-mannose, fructose, lactose, and l-rhamnose. The following substances, such as l-alanine, l-asparagine, l-cysteine, l-glycine, l-histidine, l-hydroxyproline, l-ornithine, l-proline, l-tyrosine, and methionine, can act as sole nitrogen sources, but not for l-arginine, l-glutamine, l-leucine, l-phenylalanine, l-serine, and l-valine. The cell wall diamino acid contains *meso*-diaminopimelic acid, and the whole-cell sugars contain galactose, ribose, and xylose. The main menaquinones are MK-9(H_2_), MK-9(H_4_), and MK-9(H_6_). The polar lipid profile contains diphosphatidylglycerol, phosphatidylcholine, phosphatidylethanolamine, phosphatidylglycerol, and phosphatidylinositol. The major fatty acids (>10%) were *iso*-C_16:0_, C_16:0_, and C_17:1_ ω6c.

The type strain, HUAS 11-8^T^ (= JCM 35980^T^ = MCCC 1K08337^T^), was isolated from the rhizosphere soil of *Cynara scolymus* collected in Changde city, Hunan Province, China. The DNA G + C content of the type strain genome is 70.3%. The GenBank/EMBL/DDBJ accession number for the 16S rRNA gene sequence is OQ363138. The whole-genome shotgun sequence has been deposited at DDBJ/ENA/GenBank under the accession code CP113836.

### 4.2 Emended description of *Amycolatopsis echigonensis*

Later heterotypic synonym: *Amycolatopsis niigatensis* Ding et al. (2007) (Approved Lists 2007).

The description is as before, with the following modifications. The DNA G + C content of the type strain genome is 69.5%, its approximate size is 9.66 Mbp, and its GenBank accession number is NZ_JACJHR000000000.

The type strain is LC2^T^ (=IAM 15387^T^ = CCTCC AB206019^T^ = DSM 45164^T^ = JCM 21831^T^).

## Data availability statement

The datasets presented in this study can be found in online repositories. The names of the repository/repositories and accession number(s) can be found in the article/Supplementary material.

## Author contributions

AD: Conceptualization, Investigation, Writing–original draft. LF: Writing–review & editing, Resources, Methodology. PM: Writing–review & editing. YZ: Writing–original draft, Data curation, Formal analysis. TT: Writing–original draft, Investigation, Software. JG: Visualization, Writing – review & editing.

## Footnotes

^1^ https://lpsn.dsmz.de/genus/*Amycolatopsis*

^2^ https://www.ezbiocloud.net/

^3^ https://rast.nmpdr.org

^4^ https://antismash.secondarymetabolites.org

^5^ https://card.mcmaster.ca

^6^ https://crisprcas.i2bc.paris-saclay.fr

^7^ http://www.pathogenomics.sfu.ca/islandviewer
