# Supplementary material for: New insights into the relationship between the average nucleotide identity and the digital DNA–DNA hybridization values in the genus Amycolatopsis and Amycolatopsis cynarae sp. nov., a novel actinobacterium from the rhizosphere soil of Cynara scolymus, and proposal of Amycolatopsis niigatensis as a synonym of Amycolatopsis echigonensis based on comparative genomic analysis
Source: Front Microbiol. 2024 Apr 15;15:1359021. doi: 10.3389/fmicb.2024.1359021 (PMC11056570; doi:10.3389/fmicb.2024.1359021)
Supplement: Supplementary file 1 [file Data_Sheet_1.docx]

Supplementary materials

Corresponding relationship between the average nucleotide identity and the digital DNA-DNA hybridization values in the genus *Amycolatopsis* and *Amycolatopsis cynarae* sp. nov., a novel actinobacterium from the rhizosphere soil of *Cynara scolymus* and proposal of *Amycolatopsis niigatensis* as a synonym of *Amycolatopsis echigonensis* based on comparative genomic analysis

Aihua Deng†^1^, Li Fu†^2^, Ping Mo^1^, Yaxi Zheng^1^, Ting Tang^1^, Jian Gao^*2^

Author affiliations:

^1^Key Laboratory of Agricultural Products Processing and Food Safety in Hunan Higher Education, Hunan Provincial Engineering Research Center for Fresh Wet Rice Noodles, Science and Technology Innovation Team for Efficient Agricultural Production and Deep Processing at General University in Hunan Province, Hunan Provincial Key Laboratory for Health Aquaculture and Product Processing in Dongting Lake Area, Hunan Provincial Key Laboratory for Molecular Immunity Technology of Aquatic Animal Diseases, State Key Laboratory of Developmental Biology of Freshwater Fish, College of Life and Environmental Sciences, Hunan University of Arts and Science, Changde 415000, Hunan Province, China.

^2^School of Life and Health Sciences, Hunan University of Science and Technology, Xiangtan 411201, Hunan Province, China.

†These authors contributed equally to this work.

*Correspondence: Jian Gao, xtgojian@hnust.edu.cn.

**Fig.S1** The correlations between ANIb and dDDH from the 29 pairs of *Amycolatopsis* species

**Fig.S2.** Neighbor-joining phylogenetic tree based on 16S rRNA gene sequences showing the relationship between selected species of the genus *Amycolatopsis*. *Nocardia farcinica* NBRC 15532^T^ was used as an outgroup. Bootstrap percentages over 50 % derived from 1000 replications are showed at the nodes. Dots indicate branches also recovered in the neighbor-joining and maximum-parsimony trees. Bar, 0.010 substitutions per site.

*Amycolatopsis coloradensis* DSM 44225^T^ (NR_114859.1)

*Amycolatopsis umgeniensis* UM16^T^ (NR_115688.1)

*Amycolatopsis keratiniphila* DSM 44409^T^ (NZ_LQMT02000063.1)

*Amycolatopsis lurida* DSM 43134^T^ (NZ_FNTA01000004.1)

*Amycolatopsis australiensis* DSM 44671^T^ (FPJG01000006)

*Amycolatopsis niigatensis* DSM 45165^T^ (NZ_PJMY01000003)

*Amycolatopsis echigonensis* LC2^T^ (NR_041404.1)

*Amycolatopsis saalfeldensis* DSM 44993^T^ (NZ_FOEF01000063.1)

*Amycolatopsis magusensis* DSM 45510^T^ (NR_109060.1)

*Amycolatopsis cihanbeyliensis* BNT52^T^ (NR_109530.1)

*Amycolatopsis jiangsuensis* KLBMP 1262^T^ (NR 109638.1)

*Amycolatopsis anabasis* EGI 650086^T^ (NR_170415.1)

*Amycolatopsis nigrescens* CSC17Ta-90^T^ (NR_043880.1)

*Amycolatopsis sacchari* K24^T^ (NR_028774.1)

***Amycolatopsis* sp. HUAS 11-8^T^ (OQ363138.1)**

*Amycolatopsis rhizosphaerae* TBRC 6029^T^ (NR_180744.1)

*Amycolatopsis acidicola* K81G1T (NR_176551.1)

*Amycolatopsis acidiphila* JCM 30562^T^ (MN399888.1)

*Amycolatopsis bartoniae* DSM 45807^T^ (MN399889.1)

*Nocardia farcinica* NBRC 15532^T^ (NZ_BDBJ01000178)

100

100

100

97

56

99

52

98

87

60

50

0.010

**Fig.S3.** Maximum-parsimony phylogenetic tree based on 16S rRNA gene sequences showing the relationship between selected species of the genus *Amycolatopsis*. *Nocardia farcinica* NBRC 15532^T^ was used as an outgroup. Bootstrap percentages over 50 % derived from 1000 replications are showed at the nodes. Dots indicate branches also recovered in the neighbor-joining and maximum-parsimony trees.

***Amycolatopsis* sp. HUAS 11-8^T^ (OQ363138.1)**

*Amycolatopsis rhizosphaerae* TBRC 6029^T^ (NR_180744.1)

*Amycolatopsis sacchari* K24^T^ (NR_028774.1)

*Amycolatopsis anabasis* EGI 650086^T^ (NR_170415.1)

*Amycolatopsis cihanbeyliensis* BNT52^T^ (NR_109530.1)

*Amycolatopsis jiangsuensis* KLBMP 1262^T^ (NR_109638.1)

*Amycolatopsis nigrescens* CSC17Ta-90^T^ (NR_043880.1)

*Amycolatopsis australiensis* DSM 44671^T^ (FPJG01000006)

*Amycolatopsis coloradensis* DSM 44225^T^ (NR_114859.1)

*Amycolatopsis umgeniensis* UM16^T^ (NR_115688.1)

*Amycolatopsis keratiniphila* DSM 44409^T^ (NZ_LQMT02000063.1)

*Amycolatopsis lurida* DSM 43134^T^ (NZ_FNTA01000004.1)

*Amycolatopsis niigatensis* DSM 45165^T^ (NZ_PJMY01000003)

*Amycolatopsis echigonensis* LC2^T^ (NR_041404.1)

*Amycolatopsis saalfeldensis* DSM 44993^T^ (NZ_FOEF01000063.1)

*Amycolatopsis magusensis* DSM 45510^T^ (NR_109060.1)

*Amycolatopsis acidicola* K81G1^T^ (NR_176551.1)

*Amycolatopsis acidiphila* JCM 30562^T^ (MN399888.1)

*Amycolatopsis bartoniae* DSM 45807^T^ (MN399889.1)

*Nocardia farcinica* NBRC 15532^T^ (NZ_BDBJ01000178)

84

99

98

55

92

100

96

51

100

**Fig.S4.** Polar lipids composition of strain HUAS 11-8^T^.

The plate dotted with sample was subjected to two-dimensional development, with the first solvent of chloroform-methanol-water (65:25:4, v/v/v) followed by the second solvent of chloroform-methanol-acetic acid-water (80:18:12:5, v/v/v/v).

Note: Molybdophosphoric acid, molybdenum blue reagent, anisaldehyde and ninhydrin were used to detect total lipids, phospholipids, phosphatidylinositol mannosides and aminolipids respectively. A, Molybdophosphoric acid (for total lipids); B, Molybdenum blue reagent (for phospholipids); C, Anisaldehyde (for phosphatidylinositol mannosides); D, Ninhydrin (for aminolipids). DPG, diphosphatidylglycerol; PG, phosphatidylglycerol; PE, phosphatidyl ethanolamine; PC, Phosphatidylcholine; PI, phosphotidylinositol.


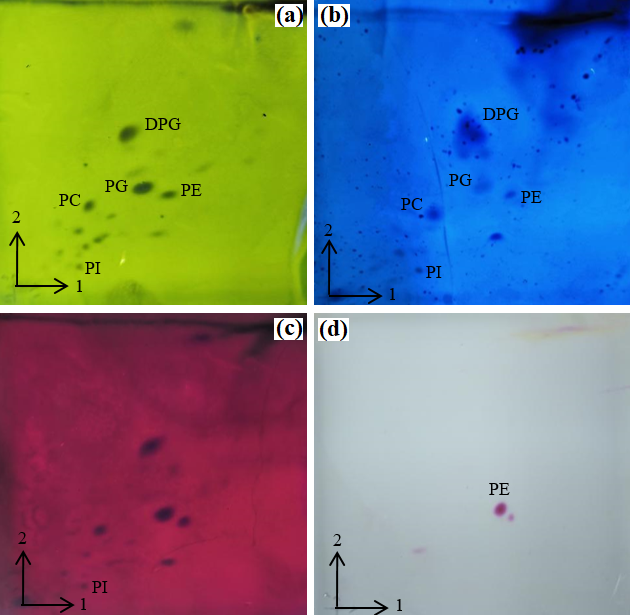


**Table S1** Quality analysis and GenBank assembly of genomes of *Amycolatopsis* species in this work

| No. | Specie | Strain | GenBank assembly | COM(%) | CON(%) | No. | Species | Strain | GenBank assembly | COM(%) | CON(%) |
| --- | --- | --- | --- | --- | --- | --- | --- | --- | --- | --- | --- |
|  | *A. alba* | DSM 44262^T^ | GCA_000384215.1 | 98.97 | 0 |  | *A. orientalis* | DSM 40040^T^ | GCA_000478275.1 | 99.16 | 0.75 |
|  | *A. balhimycina* | FH 1894^T^ | GCA_000384295.1 | 98.98 | 0.17 |  | *A. panacis* | YIM PH21725^T^ | GCA_003600245.1 | 96.70 | 3.27 |
|  | *A. coloradensis* | DSM 44225^T^ | GCA_001953865.1 | 98.66 | 1.49 |  | *A. regifaucium* | DSM 45072^T^ | GCA_900113515.1 | 99.00 | 1.52 |
|  | *A. decaplanina* | DSM 44594^T^ | GCA_000342005.1 | 98.47 | 0.58 |  | *A. rifamycinica* | DSM 46095^T^ | GCA_000695625.1 | 98.98 | 1.15 |
|  | *A. dendrobii* | DR6-1^T^ | GCA_014145675.1 | 99.06 | 2.43 |  | *A. roodepoortensis* | DSM 46661^T^ | GCA_014873915.1 | 99.00 | 4.21 |
|  | *A. echigonensis* | JCM 21831^T^ | GCA_014174495.1 | 96.71 | 2.90 |  | *A. sulphurea* | DSM 46092^T^ | GCA_002564045.1 | 97.03 | 2.52 |
|  | *A. eurytherma* | DSM 44348^T^ | GCA_003752125.1 | 99.06 | 0.44 |  | *A. thailandensis* | JCM 16380^T^ | GCA_002234405.1 | 95.12 | 0.44 |
|  | *A. japonica* | DSM 44213^T^ | GCA_000732925.1 | 98.47 | 0.65 |  | *A. thermalba* | NRRL B-24845^T^ | GCA_003385215.1 | 98.57 | 1.52 |
|  | *A. kentuckyensis* | NRRL B-24129^T^ | [GCA_002155975.1](https://www.ncbi.nlm.nih.gov/assembly/GCA_002155975.1/) | 96.19 | 0.86 |  | *A. thermoflava* | N1165^T^ | GCA_000473265.1 | 98.40 | 0 |
|  | *A. lurida* | DSM 43134^T^ | GCA_900105055.1 | 99.00 | 0.56 |  | *A. tolypomycina* | DSM 44544^T^ | GCA_900105945.1 | 98.65 | 1.1 |
|  | *A. methanolica* | 239^T^ | GCA_000371885.1 | 98.66 | 0.33 |  | *A. tucumanensis* | ABO^T^ | GCA_021654135.1 | 99.06 | 1.83 |
|  | *A. niigatensi*s | DSM 45165^T^ | GCA_002846615.1 | 98.57 | 3.46 |  | *A. vancoresmycina* | NRRL B-24208^T^ | GCA_000716785.1 | 98.84 | 0.83 |
|  | *A. nivea* | CFH S0261^T^ | GCA_004522235.1 | 99.06 | 2.10 |  | *A. vastitatis* | H5^T^ | GCA_002234595.1 | 98.59 | 1.16 |

Note: COM, Completeness; CON, Contamination.

Table S2 ANI and dDDH values of 29 pairs of *Amycolatopsis* species

| No. | Species 1 | Strain | Species 2 | Strain | ANIm(%) | ANIb(%) | dDDH(%) |
| --- | --- | --- | --- | --- | --- | --- | --- |
|  | *A. niigatensi*s | DSM 45165^T^ | *A. echigonensis* | JCM 21831^T^ | 98.74 | 98.07 | 87.9 |
|  | *A. thermoflava* | N1165^T^ | *A. eurytherma* | DSM 44348^T^ | 98.74 | 98.04 | 87.8 |
|  | *A. eurytherma* | DSM 44348^T^ | *A. methanolica* | 239^T^ | 96.64 | 95.74 | 69.3 |
|  | *A. thermoflava* | N1165^T^ | *A. methanolica* | 239^T^ | 96.62 | 95.49 | 68.7 |
|  | *A. tucumanensis* | ABO^T^ | *A. thermoflava* | N1165^T^ | 96.09 | 94.69 | 64.5 |
|  | *A. tucumanensis* | ABO^T^ | *A. methanolica* | 239^T^ | 95.54 | 94.21 | 61.8 |
|  | *A. sulphurea* | DSM 46092^T^ | *A. panacis* | YIM PH21725^T^ | 95.41 | 94.73 | 60.9 |
|  | *A. roodepoortensis* | DSM 46661^T^ | *A. lurida* | DSM 43134^T^ | 94.40 | 93.51 | 56.4 |
|  | *A. dendrobii* | DR6-1^T^ | *A. nivea* | CFH S0261^T^ | 93.85 | 92.62 | 52.5 |
|  | *A. balhimycina* | FH 1894^T^ | *A. vastitatis* | H5^T^ | 93.49 | 91.39 | 51.3 |
|  | *A. japonica* | DSM 44213^T^ | *A. decaplanina* | DSM 44594^T^ | 92.81 | 91.49 | 48.5 |
|  | *A. alba* | DSM 44262^T^ | *A. thailandensis* | JCM 16380^T^ | 92.79 | 91.19 | 48.2 |
|  | *A. niigatensi*s | DSM 45165^T^ | *A. nivea* | CFH S0261^T^ | 92.78 | 90.88 | 47.6 |
|  | *A. dendrobii* | DR6-1^T^ | *A. echigonensis* | JCM 21831^T^ | 92.65 | 90.55 | 47.2 |
|  | *A. nivea* | CFH S0261^T^ | *A. echigonensis* | JCM 21831^T^ | 92.62 | 90.56 | 47.0 |
|  | *A. thermoflava* | N1165^T^ | *A. thermalba* | NRRL B-24845^T^ | 92.25 | 90.56 | 45.5 |
|  | *A. methanolica* | 239^T^ | *A. thermalba* | NRRL B-24845^T^ | 92.07 | 90.21 | 45.0 |
|  | *A. orientalis* | DSM 40040^T^ | *A. regifaucium* | DSM 45072^T^ | 91.61 | 90.35 | 44.0 |
|  | *A. rifamycinica* | DSM 46095^T^ | *A. tolypomycina* | DSM 44544^T^ | 91.70 | 90.03 | 43.1 |
|  | *A. alba* | DSM 44262^T^ | *A. roodepoortensis* | DSM 46661^T^ | 91.15 | 89.29 | 41.7 |
|  | *A. kentuckyensis* | NRRL B-24129^T^ | *A. vancoresmycina* | NRRL B-24208^T^ | 90.89 | 88.72 | 40.6 |
|  | *A. alba* | DSM 44262^T^ | *A. coloradensis* | DSM 44225^T^ | 90.84 | 89.23 | 40.8 |
|  | *A. rifamycinica* | DSM 46095^T^ | *A. vancoresmycina* | NRRL B-24208^T^ | 90.63 | 88.78 | 39.0 |
|  | *A. tolypomycina* | DSM 44544^T^ | *A. vancoresmycina* | NRRL B-24208^T^ | 90.57 | 88.23 | 38.7 |
|  | *A. japonica* | DSM 44213^T^ | *A. coloradensis* | DSM 44225^T^ | 90.42 | 88.26 | 39.4 |
|  | *A. thailandensis* | JCM 16380^T^ | *A. orientalis* | DSM 40040^T^ | 90.41 | 88.37 | 39.5 |
|  | *A. kentuckyensis* | NRRL B-24129^T^ | *A. vastitatis* | H5^T^ | 90.27 | 87.38 | 38.5 |
|  | *A. lurida* | DSM 43134^T^ | *A. orientalis* | DSM 40040^T^ | 90.12 | 88.20 | 38.8 |
|  | *A. rifamycinica* | DSM 46095^T^ | *A. vastitatis* | H5^T^ | 90.07 | 87.49 | 37.7 |

Table S3 Cultural characteristics of strain HUAS 11-8^T^ and *Amycolatopsis rhizosphaerae* JCM 32589^T^.

| Characteristics | 1 | 2 |
| --- | --- | --- |
| Color of aerial mycelium on Reasoner' 2A | Green | Gray |
| Color of aerial mycelium on Reasoner' 2A | White | White |
| Diffusible pigment on Reasoner' 2A | None | None |
| Color of aerial mycelium on No. 1 | White | White |
| Color of aerial mycelium on No. 1 | White | White |
| Diffusible pigment on No. 1 | None | None |
| Color of aerial mycelium on ISP 2 | Gray | White |
| Color of substrate mycelium on ISP 2 | Green | Yellow |
| Diffusible pigment on ISP 2 | None | None |
| Color of aerial mycelium on ISP 3 | White/Gray | None |
| Color of substrate mycelium on ISP 3 | White | None |
| Diffusible pigment on ISP 3 | None | None |
| Color of aerial mycelium on ISP 4 | White | None |
| Color of substrate mycelium on ISP 4 | White | None |
| Diffusible pigment on ISP 4 | None | None |
| Color of aerial mycelium on ISP 5 | Gray | White |
| Color of substrate mycelium on ISP 5 | Gray | Yellow |
| Diffusible pigment on ISP 5 | None | None |
| Color of aerial mycelium on ISP 6 | Yellow | Light magenta |
| Color of substrate mycelium on ISP 6 | Yellow | Light magenta |
| Diffusible pigment on ISP 6 | None | None |
| Color of aerial mycelium on ISP 7 | Gray | White |
| Color of substrate mycelium on ISP 7 | Gray | White |
| Diffusible pigment on ISP 7 | None | None |

Note: 1, strain HUAS 11-8^T^; 2, *Amycolatopsis rhizosphaerae* JCM 32589^T^; No. 1, Gause's synthetic No. 1 medium. All data were from this study.

Table S4 The fatty acids composition of HUAS 11-8^T^ and *Amycolatopsis rhizosphaerae* JCM 32589^T^.

| Fatty acids | 1 | 2 | Fatty acids | 1 | 2 |
| --- | --- | --- | --- | --- | --- |
| C_10:0_ | tr | – | anteiso-C_16:0_ | 0.6 | tr |
| iso-C_10:0_ | tr | tr | iso-C_16:0_ | **30.5** | **37.8** |
| anteis*o*-C_11:0_ | tr | – | C_16:0_ | **10.8** | 4.9 |
| iso-C_11:0_ 3 OH | – | tr | iso-C_16:1_ G | 4.4 | 5.8 |
| iso-C_12:0_ | – | tr | iso-C_17:0_ | 3.8 | 1.4 |
| C_13:0_ | tr | – | anteiso-C_17:0_ | 7.0 | 3.0 |
| anteiso-C_14:0_ | tr | – | anteiso-C_17:1 ω9c_ | 0.6 | tr |
| C_14:0_ | 1.0 | tr | C_17:1 ω8c_ | 0.6 | 5.0 |
| C_14:1_ ω5c | tr | tr | C_17:1 ω6c_ | **10.0** | **17.2** |
| iso-C_14:0_ | 1.6 | 2.0 | C_17:0_ | tr | 1.6 |
| iso-C_14:0_ 3OH | tr | – | C_18:1_ ω9c | 4.0 | 1.0 |
| C_15:0_ 2OH | – | tr | C_18:0_ | 1.1 | 0.9 |
| iso-C_15:1_ G | tr | tr | Summed Feature 3 | 9.4 | **10.1** |
| iso-C_15:0_ | 4.7 | 3.3 | Summed Feature 5 | 1.4 | 0.9 |
| anteiso-C_15:0_ | 1.3 | 0.7 | Summed Feature 9 | 0.8 | tr |
| C_15:1_ ω6c | tr | 1.9 |  |  |  |

Note: 1, strain HUAS 11-8^T^; 2, *Amycolatopsis rhizosphaerae* JCM 32589^T^; tr, trace amount (<0.5%); –, not detected; Summed Feature 3, C_16:1_ ω7c/C_16:1_ ω6c; Summed feature 5, C_18:0_ ante/C_18:2_ ω6,9c; Summed feature 9, iso-C_17:1_ ω9c/C_16:0_ 10-methyl. All data were from this study.

Table S5 16S rRNA gene sequence similarity, *gyrB*-based genetic distance, *recN*-based genetic distance between *Amycolatopsis echigonensis* JCM 21831^T^ and it relatives

| Strains | [*Amycolatopsis echigonensis*](https://www.ezbiocloud.net/taxonomy?tn=Amycolatopsis%20echigonensis) JCM 21831^T^ | | |
| --- | --- | --- | --- |
|  | 16S rRNA gene  sequence similarity | *gyrB*-based genetic  distance (1297 bp) | *recN*-based genetic  distance (1228 bp) |
| *Amycolatopsis niigatensis* DSM 45165^T^ | 99.37% | 0.011 | 0.014 |
| *Amycolatopsis halotolerans* NRRL B-24428^T^ | 99.10% | 0.051 | 0.055 |
| *Amycolatopsis albidoflavus* NRRL B-24149^T^ | 98.96% | 0.036 | 0.059 |
| *Amycolatopsis rubida* NRRL B-24150^T^ | 98.82% | 0.049 | 0.052 |
| *Amycolatopsis circi* S1.3^T^ | 98.82% | 0.039 | 0.053 |
| *Amycolatopsis nivea* CFH S0261^T^ | 98.81% | 0.025 | 0.059 |
| *Amycolatopsis equina* SE (8)3^T^ | 98.73% | 0.039 | 0.053 |
| *Amycolatopsis dendrobii* DR6-1^T^ | 98.72% | 0.041 | 0.051 |
| *Amycolatopsis hippodromi* S3.6^T^ | 98.70% | 0.039 | 0.055 |

Table S6 GenBank accession numbers of the *gyrB* and *recN* genes used in this work

| Strains | GenBank accession number | |
| --- | --- | --- |
|  | *gyrB* | *recN* |
| [*Amycolatopsis echigonensis*](https://www.ezbiocloud.net/taxonomy?tn=Amycolatopsis%20echigonensis) JCM 21831^T^ | EU822892.1 | JF772782.1 |
| *Amycolatopsis niigatensis* DSM 45165^T^ | NZ_PJMY01000003.1 | NZ_PJMY01000003.1 |
|  | (2944383-2946350) | (6770045-6771829) |
| *Amycolatopsis halotolerans* NRRL B-24428^T^ | EU822895.1 | JF772784.1 |
| *Amycolatopsis albidoflavus* NRRL B-24149^T^ | EU822886.1 | JF772776.1 |
| *Amycolatopsis rubida* NRRL B-24150^T^ | EU822911.1 | JF772801.1 |
| *Amycolatopsis circi* S1.3^T^ | HQ021205.1 | JX465741.1 |
| *Amycolatopsis nivea* CFH S0261^T^ | NZ_SDLT01000004.1 | NZ_SDLT01000063.1 |
|  | (295153-297120) | (5732-7516) |
| *Amycolatopsis equina* SE (8)3^T^ | HQ021207.1 | JX465742.1 |
| *Amycolatopsis dendrobii* DR6-1^T^ | NZ_JACGZW010000009.1 | NZ_JACGZW010000029.1 |
|  | (10410-12377) | (5559-7343) |
| *Amycolatopsis hippodromi* S3.6^T^ | HQ021206.1 | JX465743.1 |
